# Remote Management of Patients with Cardiac Implantable Electronic Devices during the COVID-19 Pandemic

**DOI:** 10.3390/jcdd10050214

**Published:** 2023-05-14

**Authors:** Bettina Nagy, Ádám Pál-Jakab, Boldizsár Kiss, Gábor Orbán, Torda László Sélley, Zsigmond Dabasi-Halász, Barbara Bernadett Móka, László Gellér, Béla Merkely, Endre Zima

**Affiliations:** 1 Heart and Vascular Centre, Faculty of Medicine, Semmelweis University, 1122 Budapest, Hungary; 2 Biotronik Hungária Kft., 1124 Budapest, Hungary

**Keywords:** remote monitoring, chronic heart failure, cardiac implantable electronic devices, COVID-19

## Abstract

Remote monitoring (RM) is the newest function of cardiac implantable electronic devices (CIEDs). In our observational retrospective analysis, we aimed to assess whether telecardiology could be a safe alternative to routine outpatient examinations during the COVID-19 pandemic. The in- and outpatient visits, the number of acute cardiac decompensation episodes, the RM data from CIEDs, and general condition were examined via questionnaires (KCCQ, EQ-5D-5L). Regarding the enrolled 85 patients, the number of personal patient appearances was significantly lower in the year following the pandemic outbreak compared to the previous year (1.4 ± 1.4 and 1.9 ± 1.2, *p* = 0.0077). The number of acute decompensation events was five before and seven during lockdown (*p* = 0.6). Based on the RM data, there was no significant difference in heart failure (HF) markers (all related *p* > 0.05); only patient activity increased after restrictions were lifted compared to that before the lockdown (*p* = 0.03). During restrictions, patients reported increased anxiety and depression compared to their previous state (*p* < 0.001). There was no subjective change in the perception of HF symptoms (*p* = 0.7). Based on the subjective perception and CIED data, the quality of life of patients with CIED did not deteriorate during the pandemic, but their anxiety and depression intensified. Telecardiology may be a safe alternative to routine inpatient examination.

## 1. Introduction

Chronic heart failure (CHF) has become remarkably important as it is estimated to affect more than 37 million patients globally. It can be treated either in conservative or non-pharmaceutical ways [1]. Regardless of the treatment method, regular follow-up examinations are of great importance to prevent patient deterioration [2,3]. Cardiac implantable electronic devices (CIEDs) are the non-pharmaceutical therapy methods for heart failure (HF), including implantable cardioverter defibrillators (ICD) and cardiac resynchronization therapy (CRT) devices [4,5]. The increase in CIED implantations in the past decade has led to a greater need for patient follow-up [6]. In-office follow-ups are required every 3–12 months, but telemonitoring can extend the time between visits by securely transmitting diagnostic data from CIED to the manufacturer’s server [7,8,9]. Telemonitoring assists in tracking the three main parameter groups of CIEDs treatment:

1. system integrity (battery voltage, electrode impedance, stimulation threshold, sensing),
2. arrhythmias and their therapies (atrial fibrillation, malignant ventricular arrhythmias, effectiveness of antitachycardia pacing/shocks) [10,11], and
3. parameters of heart failure status (e.g., biventricular pacing ratio, heart rate trend, heart rate variability, thoracic impedance) [12,13,14].

Regular follow-up examinations of CHF patients proved challenging during the pandemic [15,16] and the utilization of remote monitoring (RM) among patients with implanted devices was expanded [17]. At the beginning of 2020, the numbers of cases of SARS-CoV-2 infection began to rise exponentially. Like in other countries, special actions were taken in Hungary. The March 15th decree introduced the requirement of postponing non-urgent healthcare services that do not need immediate medical attention. This was enforced until 18 June 2020. Since in-office follow-up examinations were restricted, telehealth, specifically telecardiology, gained greater attention [18].

This study aims to determine whether telecardiology can provide a secure and efficient replacement for in-office physical consultations for individuals suffering from CHF. By evaluating the efficacy and safety of telecardiology, the primary objective is to provide valuable insights into its potential as an alternative healthcare delivery method for cardiac patients.

## 2. Materials and Methods

### 2.1. Patient Population and Determination of the Study Periods

A single-center retrospective study was conducted at the Semmelweis University Heart and Vascular Center, Budapest, Hungary, between 1 January 2020 and 31 August 2020. Study participants were adult patients (≥18 years) with a CIED with a remote monitoring function. Only data from those who met all the eligibility criteria were analyzed. Exclusion criteria included (i) missing clinical data and (ii) CIED follow-up in another hospital. The study protocol was reviewed and approved by the Committee of Science and Research Ethics (approval number: 106/2020) and was in accordance with the Declaration of Helsinki. Three periods were examined: pre-pandemic (from 1 January 2020 to 15 March 2020), lockdown (from 15 March 2020 to 18 June 2020), and after the lockdown (from 19 June 2020 to 31 August 2020).

### 2.2. Practical Management of CIED Patients

The follow-ups of patients with CIEDs were based on the recommendations of the working groups of the European Society of Cardiology, the American Heart Association, and the American College of Cardiology and they were carried out according to the local protocols of our center [19,20]. After hospital discharge, patients require regular monitoring at the CIED outpatient clinics, where their clinical status and the function of the implanted device can be checked. Patients in our study visited the CIED clinic one week after their implantation procedure for post-operative follow-up and to check the device’s integrity (since most electrode dislodgements and sensing issues occur in the first few weeks after implantation [19]). Subsequently, patient follow-ups occurred every 6–12 months, depending on the type and age of the device and the patient’s clinical condition. During personal follow-up visits, a general cardiology examination and a full interrogation and programming of the device were performed by an electrophysiologist specialist. In cases where there was suspicion of CIED malfunction, urgent follow-up visits with possible additional tests were necessary.

### 2.3. Number of Outpatient Clinic Visits and Acute Heart Failures

Before the COVID-19 era, regular follow-ups of CHF patients had been conducted fully in office. On 15 March 2020, the Government of Hungary announced the postponement of care for patients who did not require immediate medical attention. Due to these regulations, most of the patients were unable to attend their scheduled follow-ups in person. Instead, they communicated via telephone with the treating physician, and doctors were able to remotely assess their condition using the T-option. When a particular patient was admitted to the clinic, their data were automatically entered into the central hospital system. This provided the opportunity to retrace the number of outpatient visits. Two periods were observed to see how the number of outpatient visits to the clinic changed: the pre-pandemic period (from 16 March 2019 to 15 March 2020) and the first year of COVID-19 (from 16 March 2020 to 15 March 2021). It should be noted that the state of emergency in Hungary was lifted on 18 June 2020, when the first wave of the epidemic subsided. However, the government declared a new state of emergency on 4 November 2020.

The increase in the number and rate of acute decompensation of HF cases is another indicator of disease deterioration. Acute decompensation is defined as an emergency case related to HF requiring immediate hospitalization. Emergency cases in the lockdown period were also compared against the pre-pandemic period to analyze whether the lack of in-office personal follow-ups resulted in change in the incidence of HF decompensations. The National e-Health Infrastructure enabled us to record acute decompensation events, even if the patients were not treated at our clinic.

### 2.4. Remote Monitoring

In order to assess safety, we analyzed CIED-measured parameters during the whole study period. Possible deterioration of CHF may be diagnosed by changes in heart failure parameters monitored and recorded by the device. These parameters include intrinsic rhythm (%), CRT pacing (%), atrial and ventricular rhythm (%), patient activity (%/day), heart rate variability (ms), malignant arrhythmia episodes, and the number of premature ventricular contraction per hour. The retrieved information was sorted into three periods mentioned above.

#### The Kansas City Cardiomyopathy Questionnaire and the EQ-5D-5L Questionnaire

In order to monitor symptoms, the general condition of patients was assessed. The patients completed three sets of two validated surveys concerning the three different periods mentioned above. Two validated tests were used. First, we used the Kansas City Cardio-myopathy Questionnaire (KCCQ) to measure the symptoms of heart failure [21]. Second, the EQ-5D-5L survey test was used to assess the patient’s general condition [22]. Surveys were filled out via telephone consultation when the patients had to describe their HF state. We kindly asked them to answer each question concerning the three periods mentioned above. The answers were both asked and recorded by our research team members (B.N., B.B.M., Z.D.-H.).

### 2.5. Data Collection

In addition to the remotely monitored parameters, data were collected from the ambulatory cards and hospital records. Demographic information was also included, such as age, gender, and comorbidities such as hypertension, diabetes, dyslipidemia, thyroid function, kidney disease, and lung disease.

#### Statistical Analysis

Data were analyzed using general descriptive statistics, Pearson’s chi-squared test, Kruskal–Wallis, ANOVA, and Wilcoxon–Friedman tests. Continuous variables were expressed as mean and standard deviation (SD), and categorical variables were expressed as numbers and percentages. All hypothesis tests were two-sided, with a significance level of *p* < 0.05. The data were recorded on data collection sheet (Microsoft Excel for Mac, Microsoft, 2022). Statistical analysis was performed using statistical software platform (IBM SPSS Statistics for Mac, IBM Corp., 2022).

## 3. Results

### 3.1. Characteristics of the Study Population

Eighty-five patients (age 68 ± 9 years, 73% male) were included in the study, thirty-five patients were excluded due to missing clinical data. Forty-one (48%) patients had CRT-P (CRT with pacemaker), twenty-three (27%) patients had CRT-D (CRT with defibrillator), and nineteen (22%) patients had ICD devices. The most common comorbidities were hypertension (66 patients, 78%), diabetes mellitus (22 patients, 26%), and dyslipidemia (39 patients, 46%) (Table 1).

### 3.2. Number of Outpatient Clinic Visits and Acute Heart Failures Prior to and during the Lockdown

**Figure 1:**
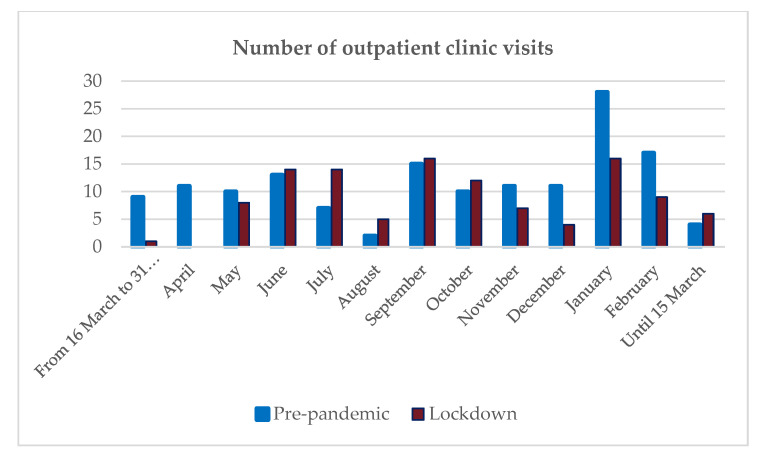
Number of outpatient clinic visits in the whole study population comparing identical months in the year before the lockdown and in the year following the outbreak of the pandemic.

During lockdown (from 15 March 2020 to 18 June 2020), the number of hospital visits decreased by 42.5% compared to the same period in the previous year (from 15 March 2020 to 18 June 2019). After the lifting of the restrictions (from 19 June 2020 to 31 August 2020), the number of hospital visits increased compensatory by 46.6% compared to the previous year’s data. In order to obtain a more detailed picture of the changes in the number of the outpatient clinic visits, we examined one year after the outbreak of the pandemic (from 15 March 2020 to 18 June 2020) and one year before the pandemic (from 19 June 2020 to 31 August 2020). The results of this analysis can be seen in Figure 1. One year prior to the lockdown period, 159 overall in-office follow-ups were registered; the number was 116 in the year following the pandemic outbreak.

There was no significant difference between the number of acute HF events in the two periods: in the year preceding the lockdown, five cases were observed, while seven cases were recorded one year after the outbreak (from 16 March 2020 to 15 March 2021) (*p* = 0.6).

### 3.3. Cardio Report from Remote Monitoring

**Figure 2:**
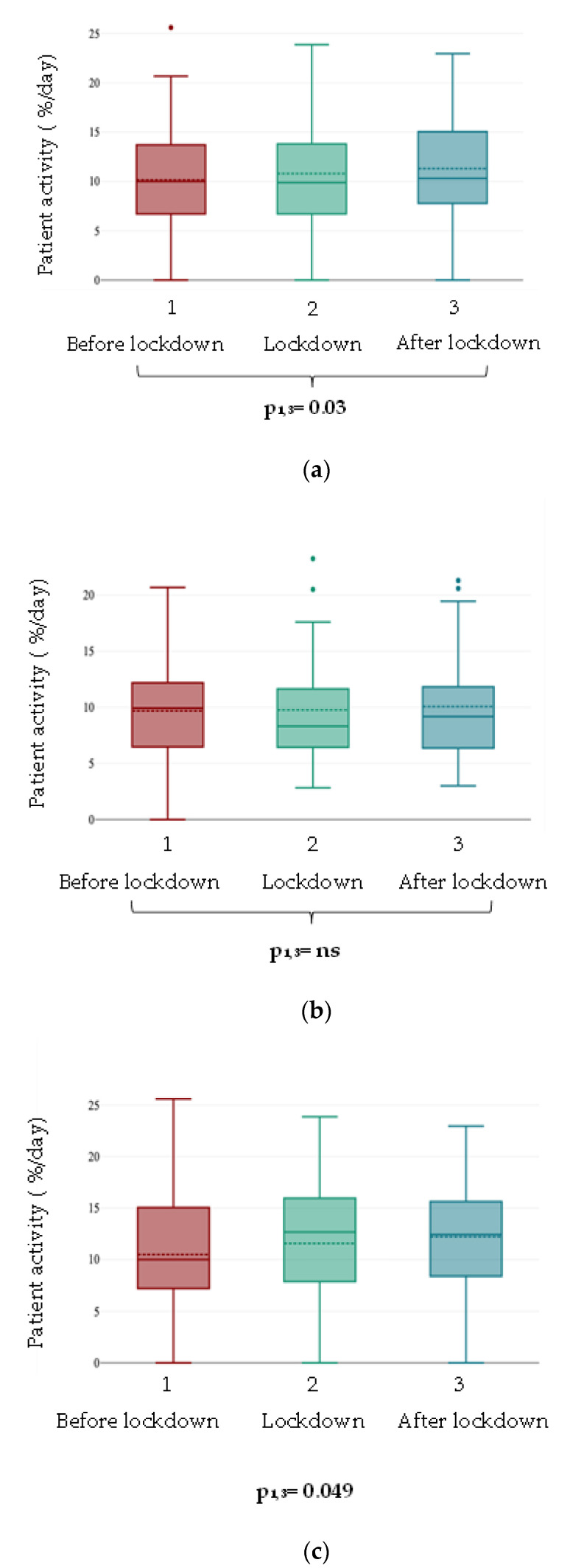
Patient activity analysis. (**a**) Total patient population. (**b**) Subgroup by age (≥70 years). (**c**) Subgroup by age (<70 years). Interquartile ranges, min, max and median values have been assessed using box plots. In Figure 2a,b. outliers are shown with colored dots according to the inspected variable. (Red: before lockdown; green: during lockdown; blue: after lockdown).

The examined remotely monitored parameters showed mostly neutral changes (Table 2). Between pre-pandemic and lockdown periods, no significant changes were found regarding most of the parameters (intrinsic rhythm, heart rate variability, number of premature heartbeats per hour, ventricular premature beat number, ventricular fibrillation episodes, ventricular tachycardia episodes; all *p* > 0.05) (Table 2). However, according to the Wilcoxon–Friedman test, patient activity significantly increased. To determine which groups had a significant difference, we conducted a Bonferroni post hoc test. There was a significant difference between the post-pandemic period compared to the pre-pandemic period (*p* = 0.03). Further subgroup analysis showed increased activity in patients under 70 years of age (*p* = 0.049), shown in Figure 2.

### 3.4. The Kansas City Cardiomyopathy Questionnaire and the EQ-5D-5L Questionnaire

Fifty-seven patients completed the questionnaires. The results are shown in Table 3. Surveys showed neutral changes concerning symptoms of CHF between the pre-pandemic and the COVID-19 period (all related *p* > 0.05). The only significant difference observed was in the responses to the mental state questions. Higher levels of anxiety and depression were reported (*p* < 0.001). CHF was reported to impact social relationships during the lockdown in a more severe way (*p* < 0.001) (for more details, see Supplementary Materials).

## 4. Discussion

The COVID-19 pandemic has led to changes in healthcare systems, including postponing non-urgent in-office care and increasing demand for hospital admissions [23]. In response to these limitations, telecardiology has garnered increased attention. Several clinical trials have evaluated the effect of telemonitoring systems on mortality. In the IN-TIME trial conducted in 2014, with 716 heart failure patients using an ICD or CRT-D device, the addition of daily remote monitoring to regular follow-ups resulted in a lower overall mortality rate at one year compared to traditional quarterly follow-ups (3.4% vs. 8.7%; *p* = 0.004) [24]. We used three methods (number of outpatient clinic visits, device parameters, and surveys) to assess the additional value of RM on the safety and effectiveness of remote monitoring of patients with CHF.

Our main findings were that the number of personal appearances in the outpatient clinic decreased significantly in the year following the pandemic outbreak (*p* = 0.0077), but the number of acute heart failure events did not change compared to the pre-lockdown period (*p* = 0.6). Patient activity increased after the restrictions were lifted (*p* = 0.03), and patients reported higher level of anxiety and depression during restrictions (*p* < 0.001) with no significant difference in subjective assessment of heart failure symptoms between pre-pandemic and lockdown periods (all *p* > 0.05).

Ziacchi and colleagues reported a higher rate of alerts, suggesting the worsening of heart failure incidence. The weekly rate of alerts was significantly higher during the lockdown (1.56 alerts/week/100 pts) and after the lockdown (1.37 alerts/week/100 pts) compared to the pre-lockdown period (0.91 alerts/week/100 pts) [25]. In contrast, we found that in-office follow-up visits significantly decreased during the pandemic due to the enactment of the lockdown (*p* = 0.0077). However, there was no significant change in the acute heart failure burden (*p* = 0.6). The absence of difference could be due to lack of power with small number of event rates. In their study, Zorzi and colleagues found no significant increase in a combined endpoint encompassing arrhythmic events and mortality among remotely monitored ICD carriers in 2020 compared to 2019 [26]. Therefore, the pandemic appears to have had a greater impact on reducing in-office follow-ups than on the incidence of acute heart failures in this patient population.

While for the most part, no differences were observed in the remote monitoring parameters transmitted by the device over the three periods studied (all related *p* > 0.05), a difference in the patients’ physical activity was detected (*p* = 0.03). However, further investigation showed that this difference was not between the period preceding the lockdown and the lockdown but between the pre-lockdown and post-lockdown periods. Contrary to this finding, several studies have reported reduced activity in patients with CHF during the lockdown. Al Fagih et al. reported that there was a 27% decline in physical activity from 2.4 to 1.8 h/day (*p* < 0.0001) [27]. In their research, Bontempi and colleagues discovered a significant decrease in both the mean and peak values of physical activity, as measured by embedded accelerometric sensors, between the period before the pandemic and during the outbreak (13% vs. 10% *p* < 0.001; 22% vs. 18%, *p* < 0.001) [28]. Bertagnin et al. screened 211 patients and found that patients’ physical activity significantly decreased in the lockdown period compared with that of the control period (active time per day 8.0% vs. 10.8%, *p* < 0.0001) [12]. Mascioli and colleagues reported similar results. Out of all the patients, 89% showed a decrease in activity. Among them, 43% had a relative reduction of 25% or more [29]. Nevertheless, in these studies, the lockdown period was not compared with the period following the end of restrictions. Meanwhile, Cunha and colleagues examined post-lockdown physical activity levels among different groups of patients based on their activity levels before, during, and after lockdown measures. The groups were categorized as non-recoverers, incomplete recoverers, recoverers, and full-recoverers. They determined that physical activity decreased during lockdown measures in all groups, and after the measures were lifted, two groups of patients did not return to their previous levels of physical activity [30]. We have also compared the period before the lockdown with the period after the end of restrictions in terms of patients’ physical activity. We found that the activity of patients significantly increased following the lifting of restrictions compared to the period preceding the lockdown. Further investigating this result, we conducted a subgroup analysis and determined that the increase in activity seen after the lifting of restrictions was observed in the age group under 70 years. The combined effect of several factors may underlie this. The increase in patient activity might be a consequence of the lockdown [31]. Patients might have consciously paid more attention to staying healthy and maintaining or even improving physical activity during the lockdown. Once the restrictions were lifted, they found it easier to resume their newly established daily exercise routine, and their physical activity increased as a result. In addition, people experienced an increased drive to participate in physical activities and exhibited improved utilization of the resources at their disposal. Monitoring changes in physical activity in CHF patients is crucial, as physical inactivity has previously been shown to significantly affect outcome in patients with CHF [30].

Our patients did not experience significant deterioration in their HF symptoms or HF-related quality of life (all related *p* > 0.05). However, we found significant differences in the patients’ psychological states between the studied periods. They reported higher levels of anxiety and depression (*p* < 0.001), and heart failure also had a negative impact on their social relationships (*p* < 0.001). This may be explained by their chronic illness, which made them more fearful and anxious about the viral infection and thus more willing to isolate themselves to avoid infection. In another study conducted during the Italian lockdown, 332 patients were enrolled in an RM program to minimize the risk of in-hospital exposure to COVID-19 infection and were divided into home and office groups to assess the efficacy of the new follow-up protocol, as well as patients’ acceptance and anxiety status. The study found that RM adoption resulted in high patient satisfaction, but in the in-office-delivered group, patients had a higher prevalence of anxiety symptoms. Of the newly enrolled RM patients, 87.5% had not reported anxiety, while 9.4% had only mild anxiety, 2.7% had moderate anxiety, and 0.7% had severe anxiety [32]. On the other hand, during lockdown, in our study, 8% of our patients struggled with severe depression and anxiety, while an additional 16% reported moderate symptoms. However, increased anxiety may not only be caused by the closures. According to Tsabedze and colleagues, more than 50% of patients who visited the CHF clinic exhibited symptoms of depression and anxiety. They suggest regular mental health screening should be conducted for patients with CHF [33]. In contrast, concerning the CHF population studied here, 83% of patients did not report any anxiety or depression symptoms regarding the period prior to the lockdown, while an additional 6% experienced only slight psychological symptoms. Mattioli and colleagues have stated that the COVID-19 pandemic has significantly increased socioeconomic stressors, which has impacted cardiovascular risk factors. In their view, physical activity can be an effective and inexpensive tool to help manage stress and depression [34].

## 5. Conclusions

The reliability of the telecardiology system is demonstrated by the fact that although the duration between in-office examinations has nearly doubled and the patients were followed-up through telecardiology, no significant changes were observed in the parameters indicating heart failure; therefore, the remote monitoring system proved to be effective. Additionally, questionnaires completed by patients indicate that their subjective assessment of heart failure symptoms did not change.

The system proved safe for CHF patients as it provided an opportunity for remote monitoring, thereby reducing the risk of infection during the pandemic. It provides adequate data on the prognosis of heart failure. The built-in event alert system allows for early detection of arrhythmia, reducing the risk of stroke by timely initiation of anticoagulant therapy and reducing the time between clinical events and decision-making.

Chronic heart failure patients maintained their health condition during this extraordinary period owing to regular online follow-ups and questionnaire-based assessments of HF status. In the future, home monitoring might gain a larger perspective of CHF patients with CIEDs.

## 6. Limitations

Our study has some limitations that should be taken into consideration. First, the sample size is relatively small, which may affect the generalizability of our findings. Consequently, the statistical power of our analysis may have been limited. Second, the impact of lockdown restrictions on patient activity levels and symptom reporting is another limitation. Patient activity levels were lower during the lockdown period, which may have led to an underestimation of the severity of heart failure symptoms such as shortness of breath and fatigue. This could have affected the validity of the results obtained from the questionnaires used in our study. Future studies should consider these limitations in order to provide a more comprehensive understanding of heart failure, particularly in different contexts.

### Open Problems

The widespread implementation of remote monitoring in clinical practice faces several challenges. First, workflow and data management standardization are needed to ensure the effective utilization of RM data by various healthcare professionals. Determining the reaction time is also a crucial factor. Reaction time is the period between receiving an alert or notification and the physician’s response to the issue. Usually, a personal examination occurs within 24 h after the alert; however, this timeframe can vary based on the physician, the patient’s condition, and the severity of the problem [35]. Second, there are economic concerns regarding the cost of technology and human resources for data management in healthcare models. Additionally, legal issues related to physician response time, patient consent, and data confidentiality need to be addressed for successful RM implementation [36].

## 7. Future Research Directions

This study highlights the need for further research to advance the management of cardiovascular diseases and conditions. One crucial area is the identification of factors that can predict acute decompensation events with the aim of improving prevention and management. Additionally, assessing the feasibility and safety of achieving optimal medication (for example, anticoagulant drug therapy) without in-office visits is essential, particularly given the growing prevalence of telemedicine. Furthermore, exploring the potential benefits of telemedicine for regular follow-ups of other cardiovascular diseases and conditions, apart from heart failure, is necessary to improve patient outcomes and reduce healthcare costs. Addressing these research directions can significantly enhance our comprehension of cardiovascular disease management and provide innovative solutions to current, patient-centered healthcare challenges.

## Figures and Tables

**Table 1:** Characteristics of the study population.

Parameters	Result (*n* = 85)
Male/female, *n* (%)	62 (72.9)/23 (27.1)
Age (year ± SD)	68 ± 9.2
CRT-P/CRT-D, *n* (%)	41 (48.2)/23 (27.1)
ICD, *n* (%)	21 (24.7)
HFrEF/HFmrEF/HFpEF, *n* (%)	46 (54.1)/21 (24.7)/18 (21.2)
Hypertension, *n* (%)	66 (77.6)
Diabetes mellitus, *n* (%)	22 (25.9)
Dyslipidaemia, *n* (%)	39 (45.9)
Hypo-/hyperthyroidism, *n* (%)	8 (9.4)
Renal failure, *n* (%)	7 (8.2)
COPD, *n* (%)	5 (5.9)

Abbreviations: COPD: chronic obstructive pulmonary disease; CRT-D: cardiac resynchronization therapy with defibrillator; CRT-P: cardiac resynchronization therapy with pacemaker; ICD: implantable cardioverter defibrillator.

**Table 2:** The results of remote monitoring parameters.

Parameter	Pre-Pandemic ± SD	CI	Lockdown ± SD	CI	After Lockdown ± SD	CI	*p*-Value
Atrial pacing (%)	31.2 ± 34.4	22.99; 38.63	30.4 ± 33.2	22.99; 37.81	29.6 ± 33.2	22.29; 36.92	0.931
LV pacing impedance (ohm)	734.9 ± 257.6	675.87; 794.07	741.5 ± 254.8	684.6; 798.43	755.8 ± 287.1	692.44; 819.08	0.406
BiV pacing (%)	96.0 ± 7.2	94.36; 97.64	96.1 ± 7.5	94.43; 97.77	95.1 ± 8.9	93.12; 97.05	0.225
CRT pacing (%)	98.3 ± 2.7	97.69; 98.91	98.3 ± 2.8	97.69; 98.93	97.9 ± 3.3	97.16; 98.61	0.590
LV sensing amplitudes daily mean (mV)	14.2 ± 4.9	13.05; 15.34	14.5 ± 4.7	13.2; 15.41	13.9 ± 4.9	12.68; 14.95	0.758
Mean atrial heart rate (bpm/min)	91.6 ± 52.3	79.56; 103.54	89.0 ± 50.7	77.59; 100.38	90.4 ± 52.7	78.6; 102.14	0.986
Heart rate variability (ms)	63.3 ± 28.0	56.82; 69.77	62.1 ± 28.3	55.74; 68.47	61.9 ± 29.0	55.41; 68.37	0.076
RA pacing impedance (ohm)	651.2 ± 343.4	570.17; 732.23	655.9 ± 340.6	577.23; 734.56	654.3 ± 338.6	576.13; 732.55	0.508
RV pacing impedance (ohm)	528.1 ± 121.9	500.44; 555.66	529.9 ± 129.4	500.98; 557.64	531.7 ± 136.0	503.06; 562.06	0.363
VAT stimulation (%)	61.5 ± 36.1	52.33; 68.97	62.5 ± 34.9	55.35; 70.86	61.7 ± 34.9	55.38; 70.68	0.572
VT episodes	1.8 ± 11.4	−1.26; 4.88	1.8 ± 11.4	−1.23; 4.91	1.9 ± 12.1	−1.26; 5.02	0.941
VF episodes	0.3 ± 0.9	0.08; 0.55	0.4 ± 1.0	0.14; 0.63	0.5 ± 1.2	0.15; 0.8	0.840
Patient activity (%/day)	10.2 ± 5.5	8.95; 11.35	10.8 ± 5.4	9.62; 11.98	11.3 ± 5.3	10.16; 12.46	0.013

Abbreviations: BiV: biventricular; CI: confidence interval; CRT: cardiac resynchronization therapy; LV: left ventricle; RA: right atrium; RV: right ventricle; VAT: percentage of ventricular paces that were triggered by an intrinsic atrial event; VF: ventricular fibrillation; VT: ventricular tachycardia.

**Table 3:** The results of the given responses of the Kansas City Cardiomyopathy Questionnaire and the EQ-5D-5L questionnaire.

Question	Pre-Pandemic	Lockdown	*p*-Value
How many times has fatigue limited your ability to do what you want?	5.96 ± 1.39	6.13 ± 1.25	0.260
How much has your fatigue bothered you?	5.07 ± 1.14	5.11 ± 1.25	0.814
How much has your shortness of breath bothered you?	5.45 ± 1.23	5.60 ± 0.79	0.510
How many times did you have swelling in your feet, ankles or legs when you woke up in the morning?	4.55 ± 0.99	4.71 ± 0.74	0.455
How much does your heart failure affect your lifestyle?	4.63 ± 0.93	4.68 ± 0.87	0.582
How much does your heart failure affect your mobility?	1.40 ± 1.18	1.31 ± 1.14	0.090
How much does heart failure affect visiting family or friends outside of your home?	4.85 ± 0.84	5.27 ± 0.99	<0.001
How often have you felt anxiety or depression?	1.33 ± 0.75	1.65 ± 0.97	<0.001

The table contains questions related to the most common symptoms described in the 2021 European Society of Cardiology guidelines and psychological state [1]. Further details can be found in the Supplementary Materials.

## Data Availability

The data presented in this study are available on request from the corresponding author (zima.endre@gmail.com).

## Supplementary Materials

The following supporting information can be downloaded at: https://www.mdpi.com/article/10.3390/jcdd10050214/s1, Supplementary Materials, The Kansas City Cardiomyopathy Questionnaire.
