# Supplementary material for: Remote Management of Patients with Cardiac Implantable Electronic Devices during the COVID-19 Pandemic
Source: J Cardiovasc Dev Dis. 2023 May 14;10(5):214. doi: 10.3390/jcdd10050214 (PMC10219157; doi:10.3390/jcdd10050214)

Supplementary Material

The Kansas City Cardiomyopathy Questionnaire

Pre-COVID:

(1.) Heart failure affects different people in different ways. Some feel shortness of breath while others feel fatigue. Please indicate how much you are limited by heart failure (shortness of breath or fatigue) in your ability to do the following activities.

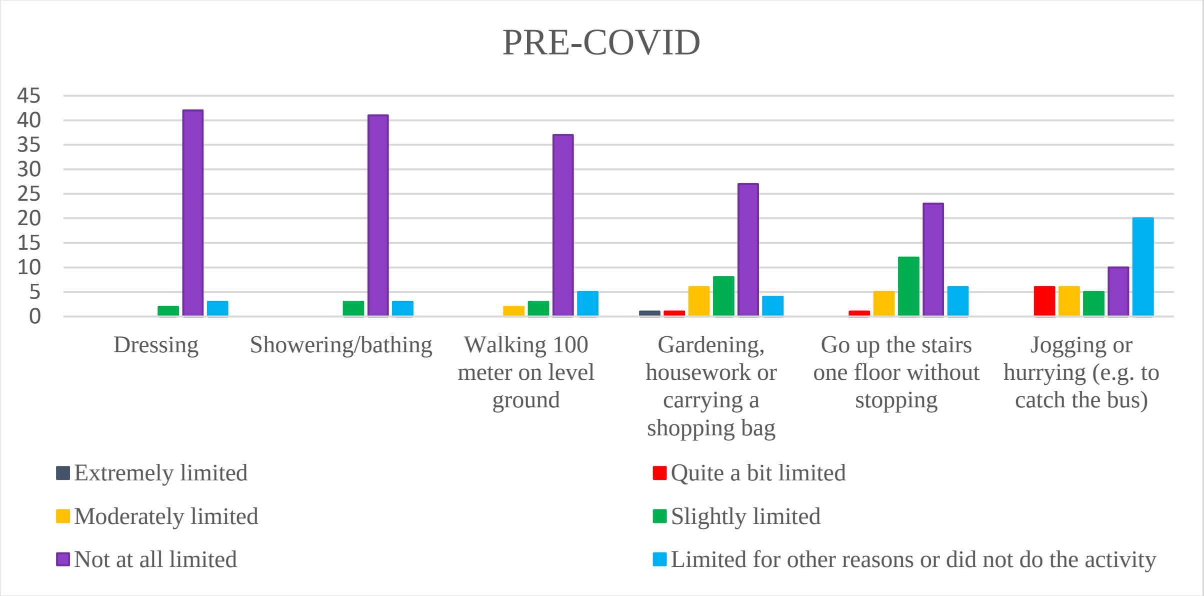

(2.) My symptoms of **heart failure** (shortness of breath, fatigue or ankle swelling) have become …

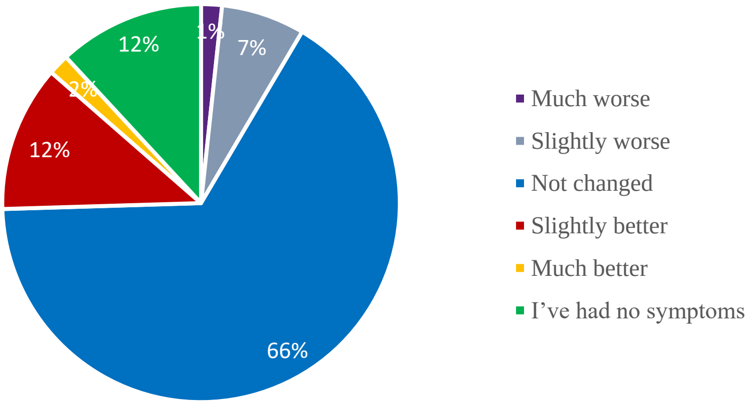

(3.) How many times did you have swelling in your feet, ankles or legs when you woke up in the morning?

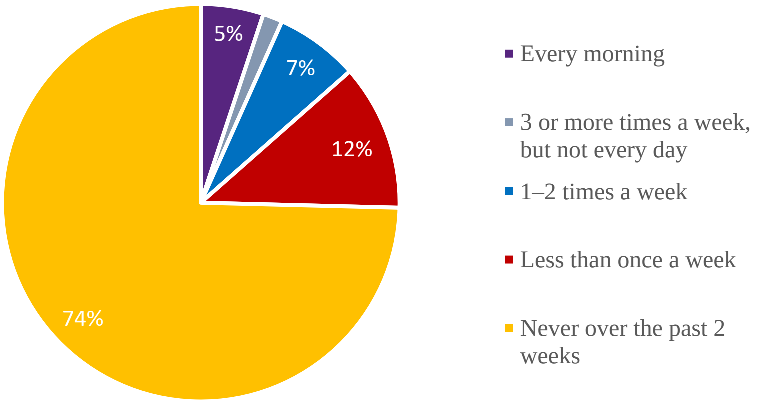

(4.) How much has **swelling** in your feet, ankles or legs bothered you? It has been …

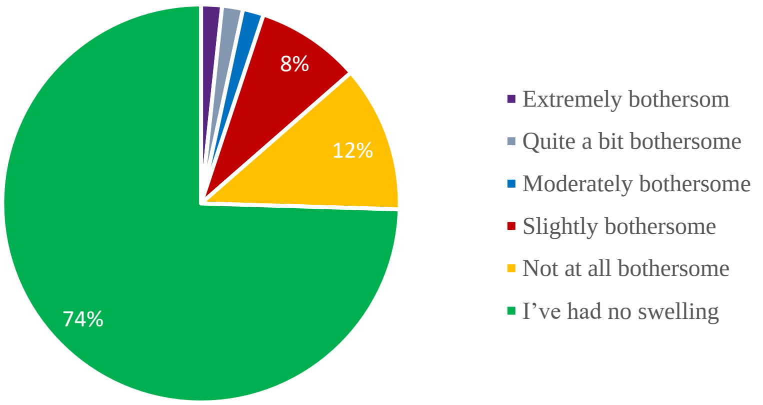

(5.) How many times has **fatigue** limited your ability to do what you want?

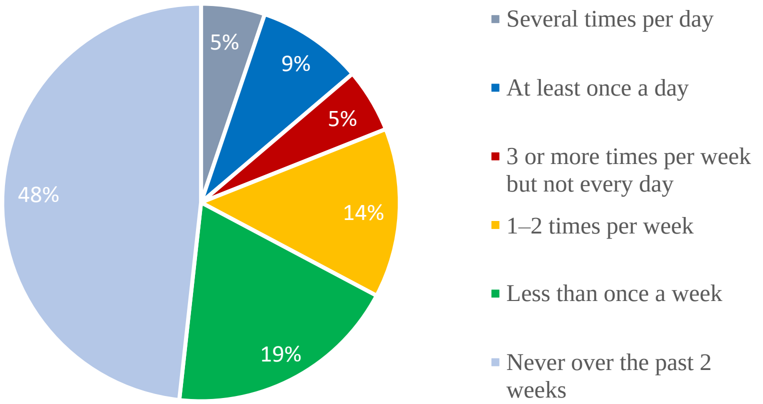

(6.) How much has your **fatigue** bothered you? It has been …

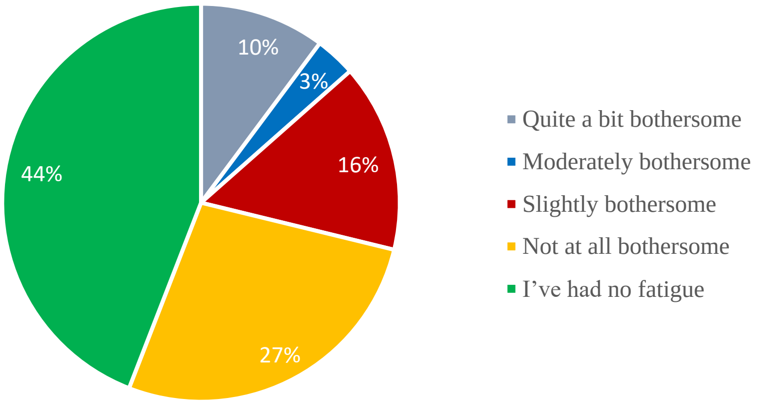

(7.) On average, how many times has **shortness of breath** limited your ability to do what you wanted?

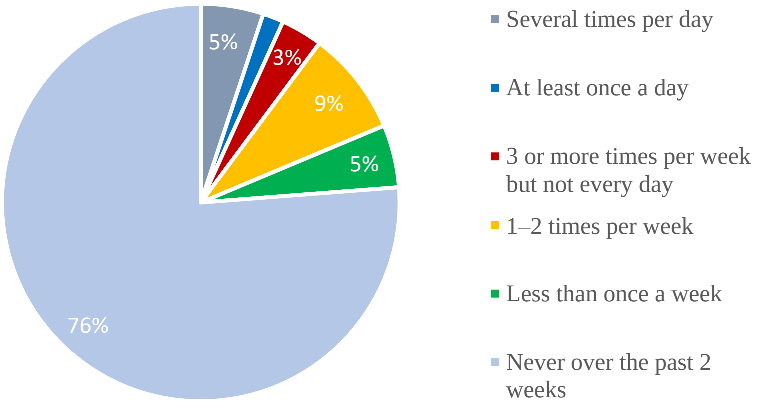

(8.) How much has your **shortness of breath** bothered you? It has been …

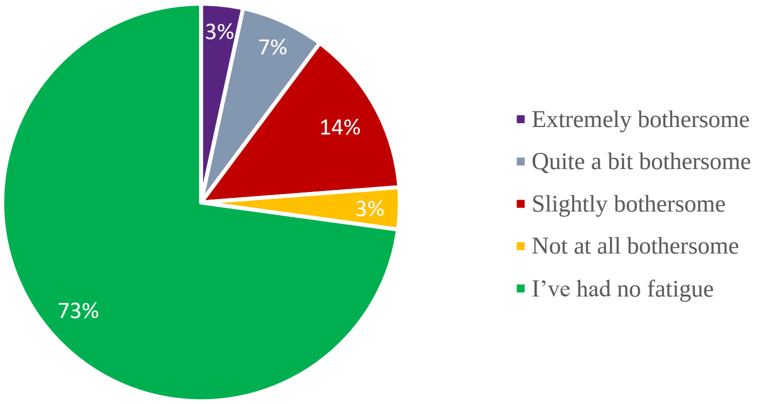

(9.) On average, how many times have you been forced to sleep sitting up in a chair or with at least 3 pillows to prop you up because of **shortness of breath**?

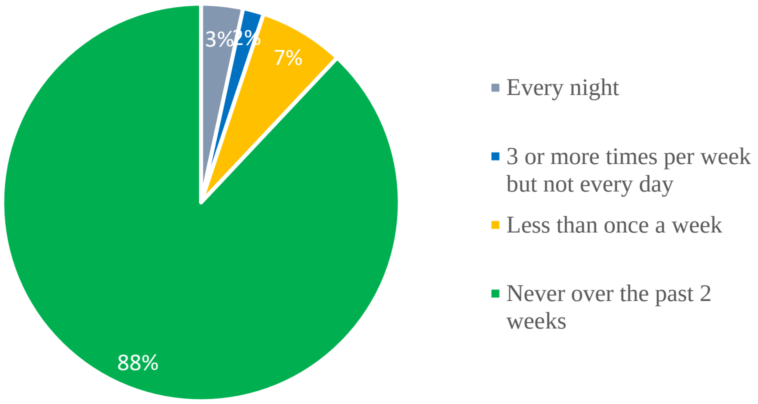

(10.) How much has your **heart failure** limited your enjoyment of life?

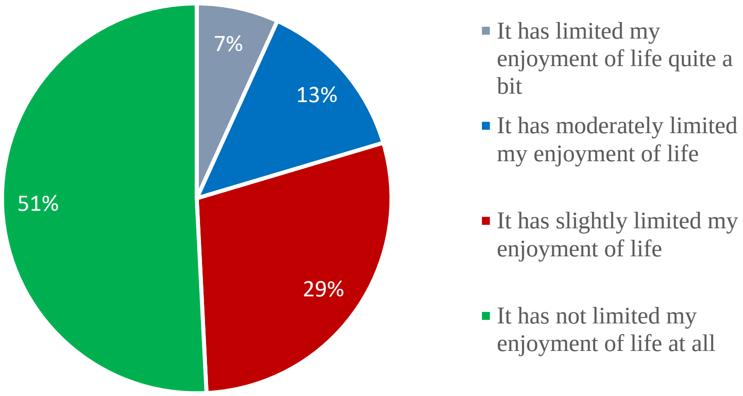

(11.) If you had to spend the rest of your life with your **heart failure** the way it was during the pre-COVID period, how would you feel about this?

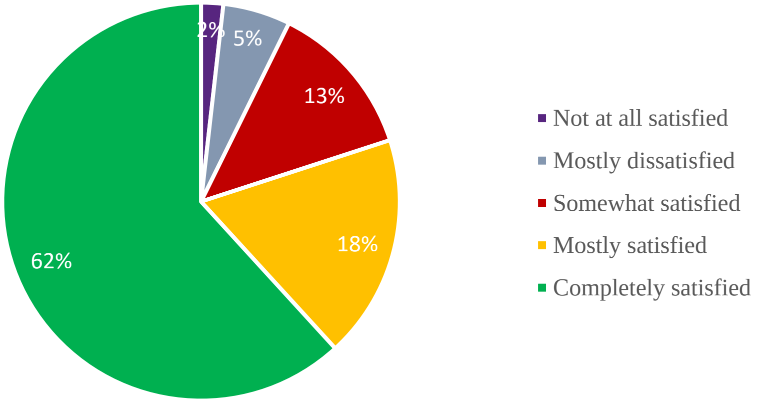

(12.) How often have you felt discouraged or down in the dumps because of your **heart failure**?

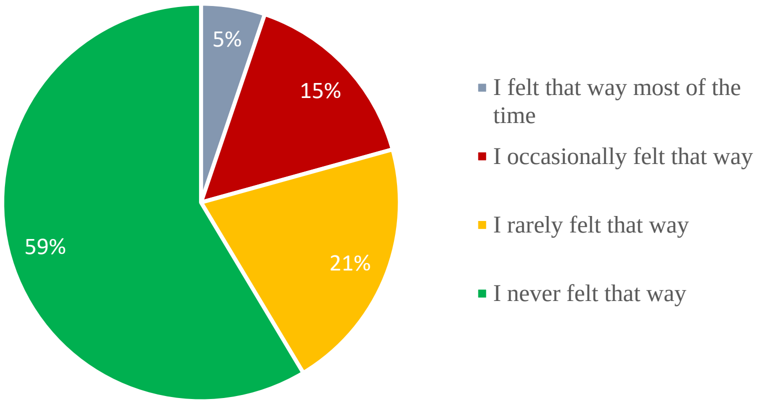

(13.) How much does your heart failure affect your lifestyle? Please indicate how your heart failure may have limited your participation in the following activities.

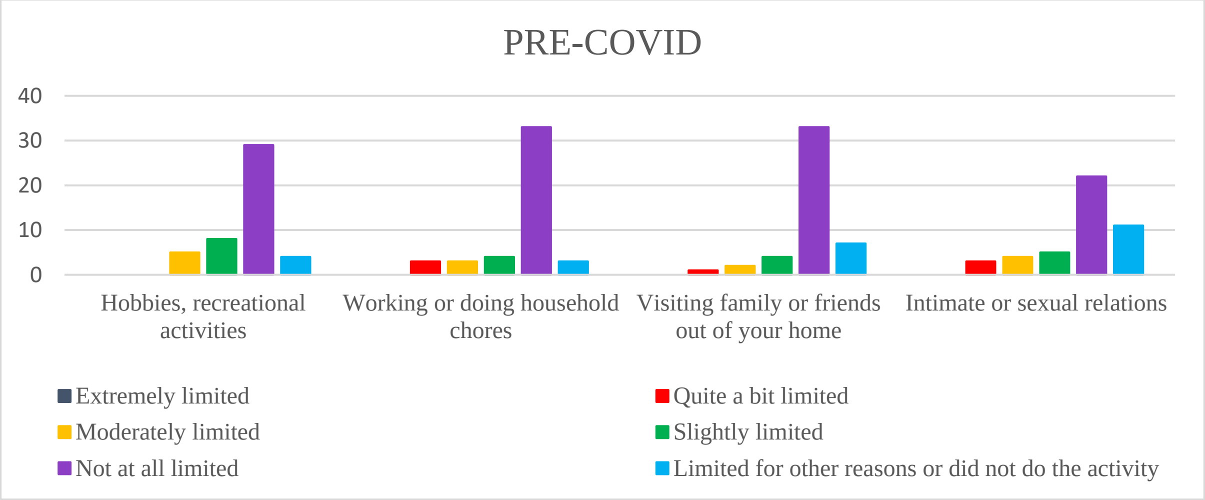

<u>Lockdown</u>

(1.) Heart failure affects different people in different ways. Some feel shortness of breath while others feel fatigue. Please indicate how much you are limited by heart failure (shortness of breath or fatigue) in your ability to do the following activities.

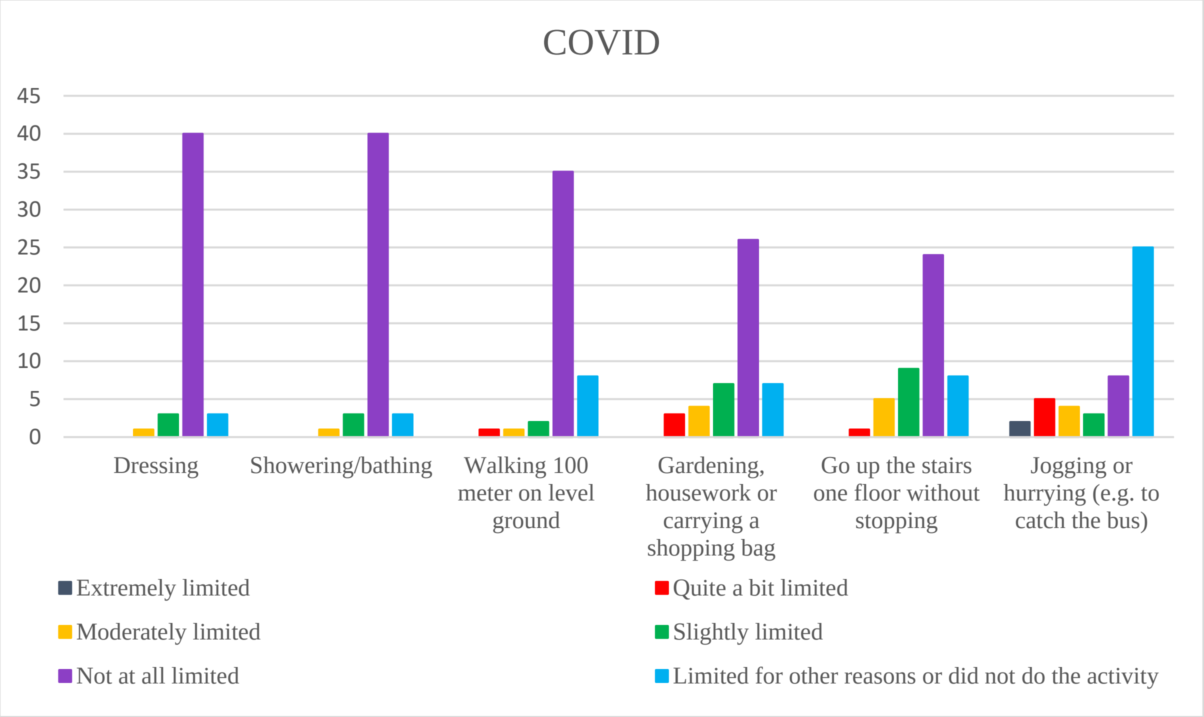

(2.) My symptoms of **heart failure** (shortness of breath, fatigue or ankle swelling) have become …

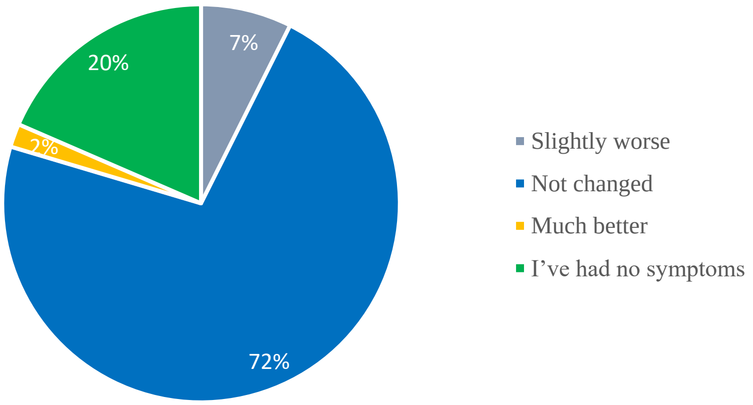

(3.) How many times did you have swelling in your feet, ankles or legs when you woke up in the morning?

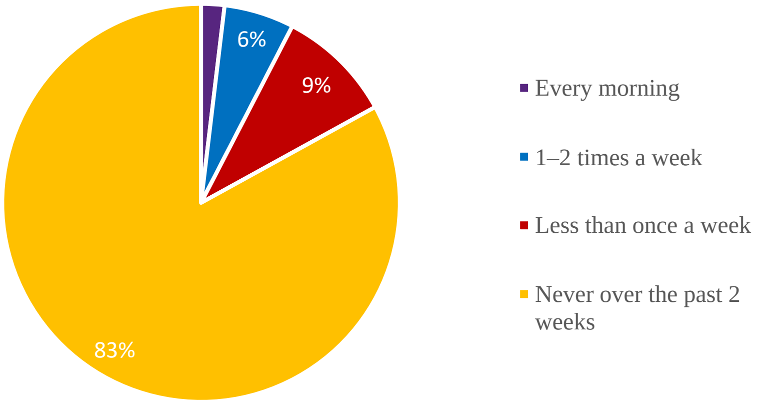

(4.) How much has **swelling** in your feet, ankles or legs bothered you? It has been …

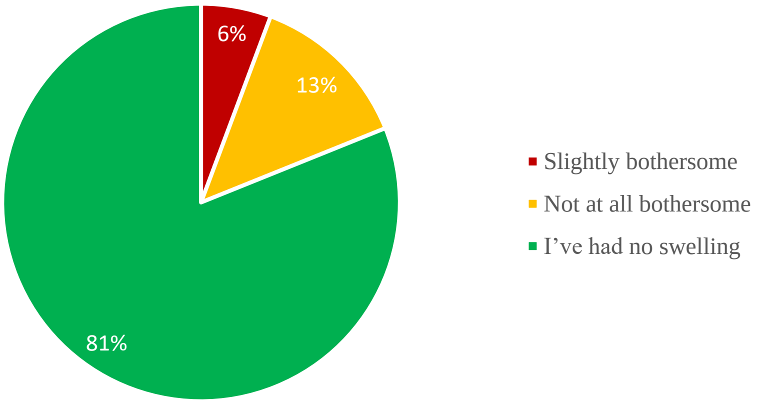

(5.) How many times has **fatigue** limited your ability to do what you want?

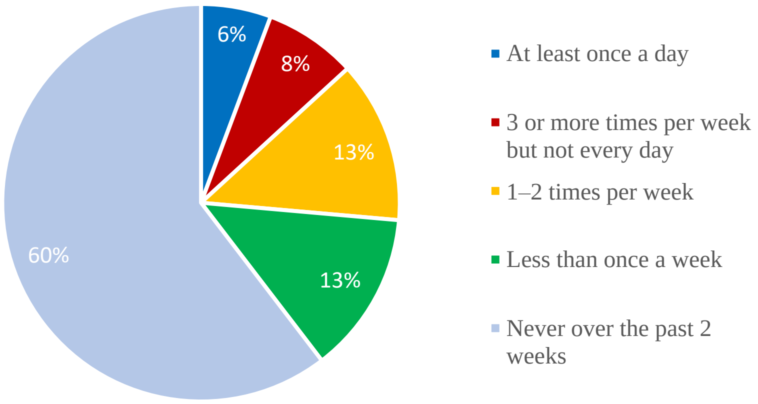

(6.) How much has your **fatigue** bothered you? It has been …

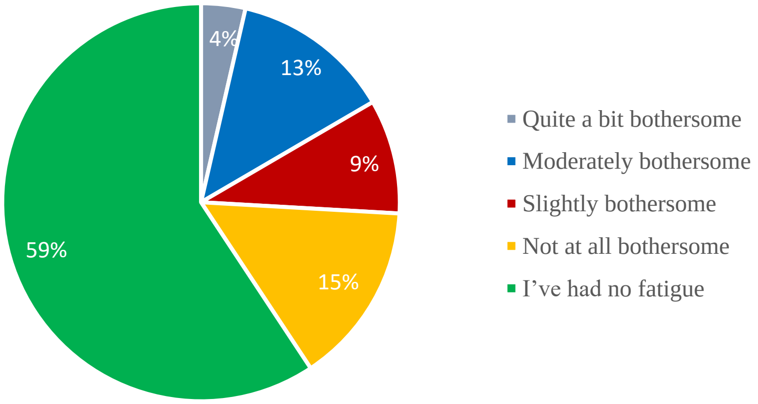

(7.) On average, how many times has **shortness of breath** limited your ability to do what you wanted?

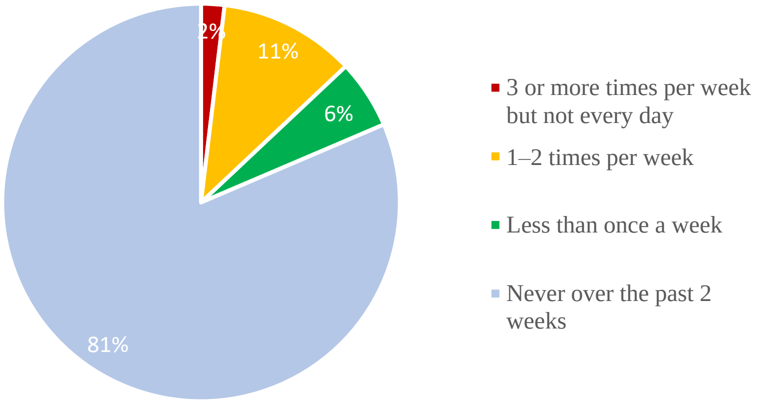

(8.) How much has your **shortness of breath** bothered you? It has been …

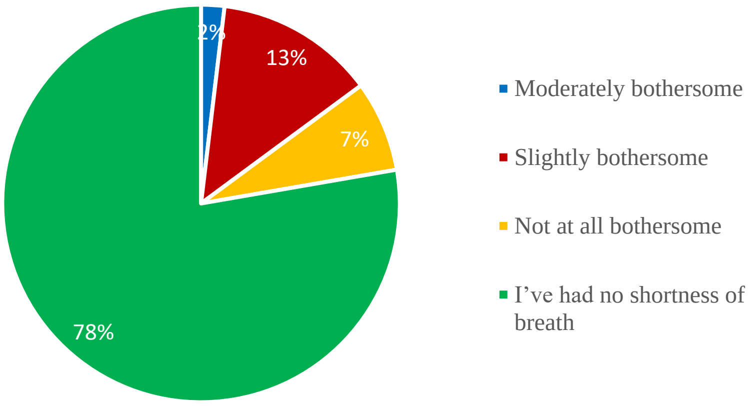

(9.) On average, how many times have you been forced to sleep sitting up in a chair or with at least 3 pillows to prop you up because of **shortness of breath**?

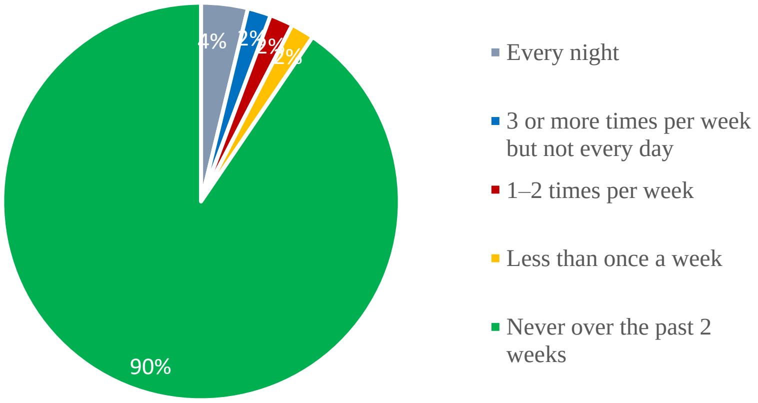

(10.) How much has your **heart failure** limited your enjoyment of life?

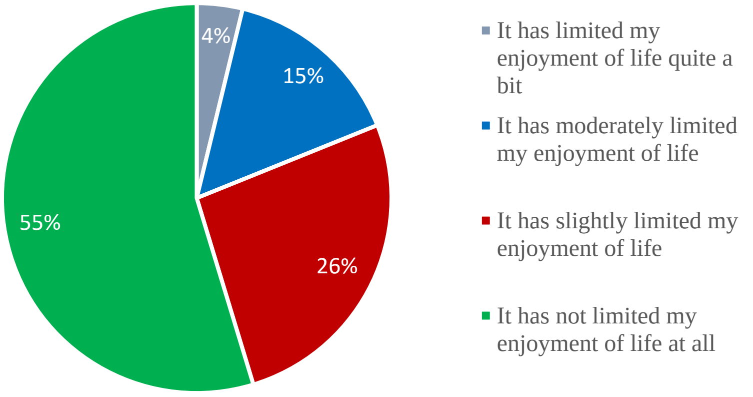

(11.) If you had to spend the rest of your life with your **heart failure** the way it was <u>during the lockdown period</u>, how would you feel about this?

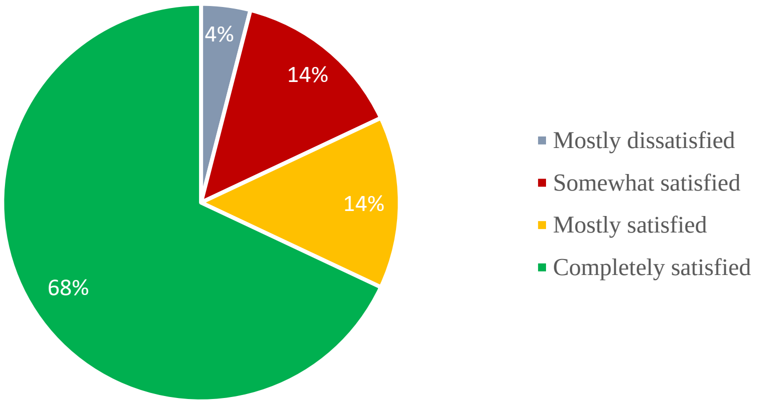

(12.) How often have you felt discouraged or down in the dumps because of your **heart failure**?

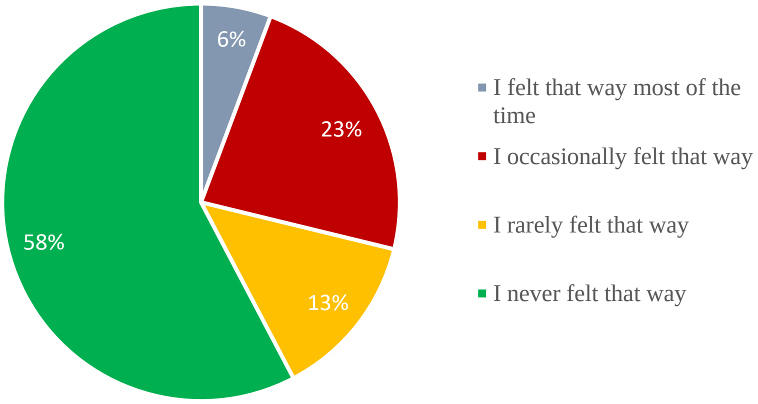

(13.) How much does your heart failure affect your lifestyle? Please indicate how your heart failure may have limited your participation in the following activities.

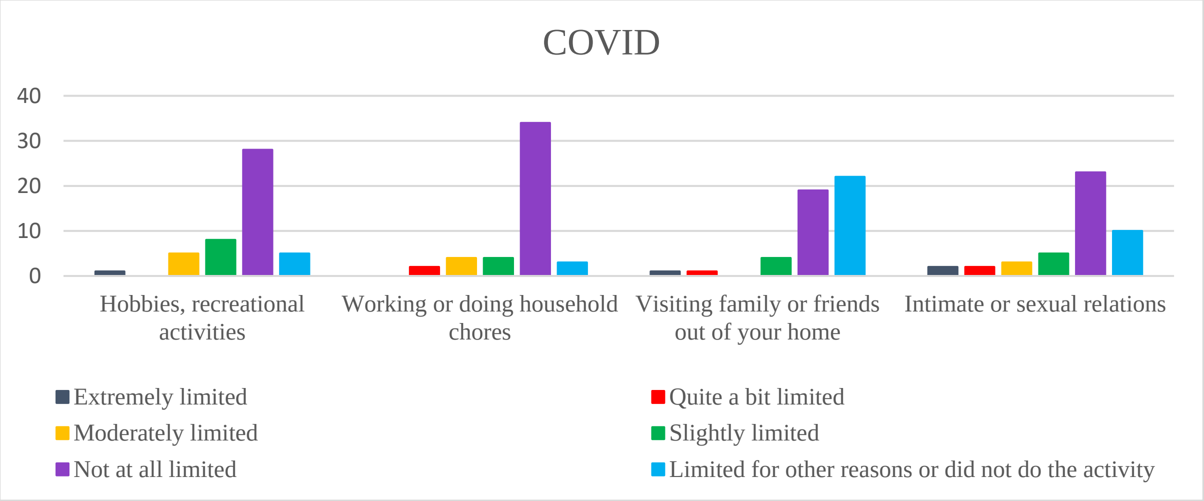

<u>Post-COVID</u>

(1.) Heart failure affects different people in different ways. Some feel shortness of breath while others feel fatigue. Please indicate how much you are limited by heart failure (shortness of breath or fatigue) in your ability to do the following activities.

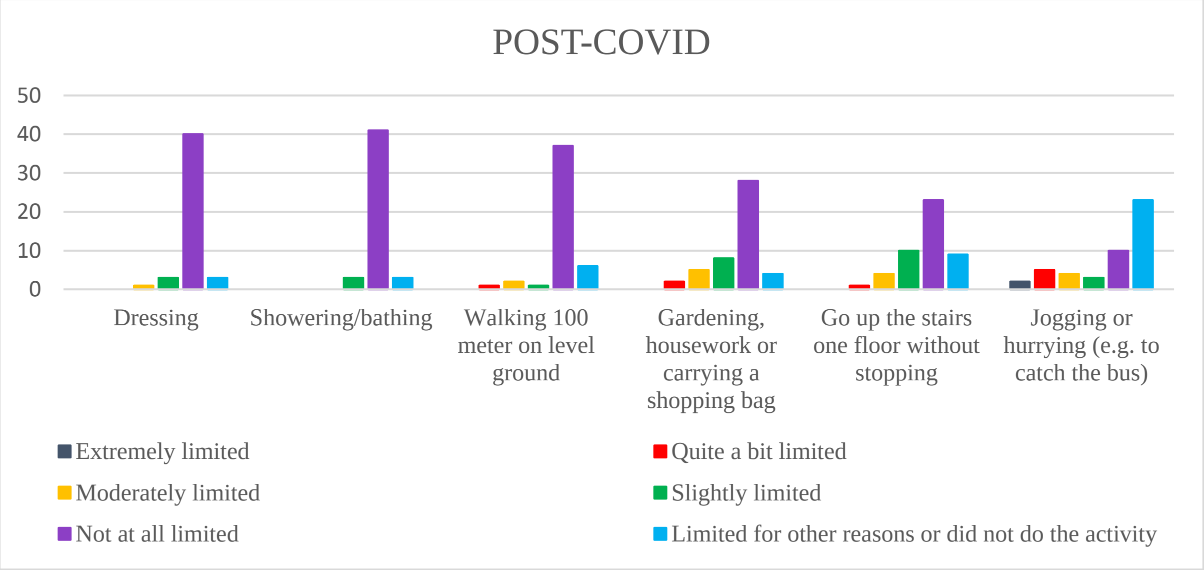

(2.) My symptoms of **heart failure** (shortness of breath, fatigue or ankle swelling) have become …

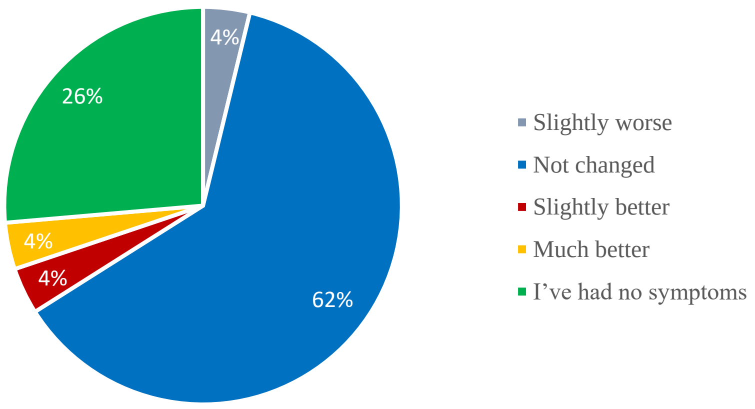

(3.) How many times did you have swelling in your feet, ankles or legs when you woke up in the morning?

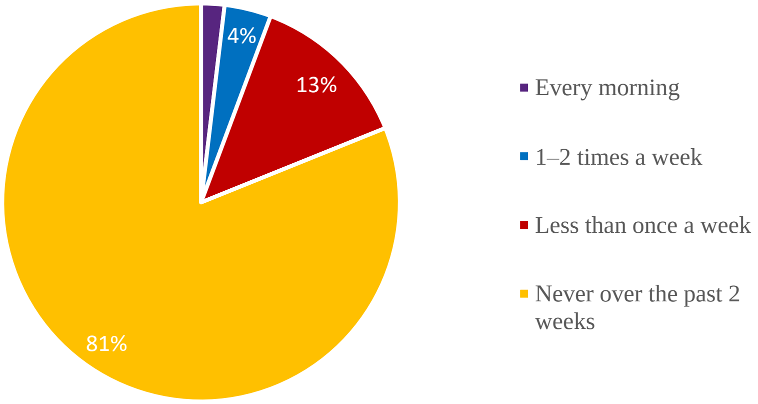

(4.) How much has **swelling** in your feet, ankles or legs bothered you? It has been …

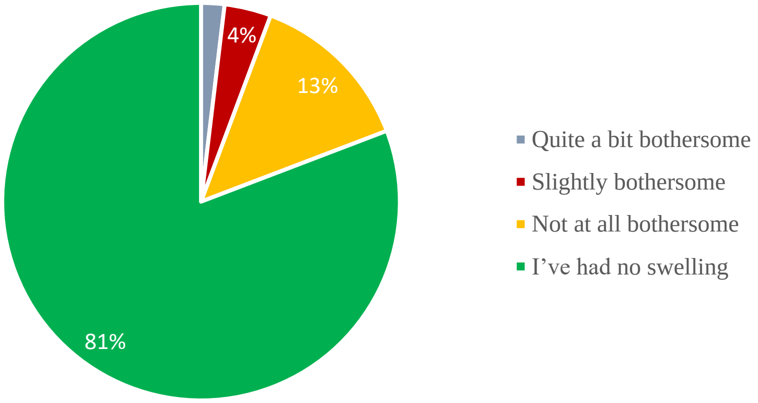

(5.) How many times has **fatigue** limited your ability to do what you want?

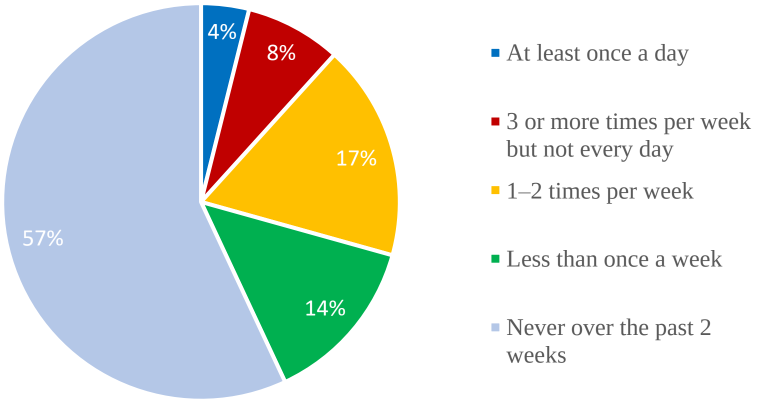

(6.) How much has your **fatigue** bothered you? It has been …

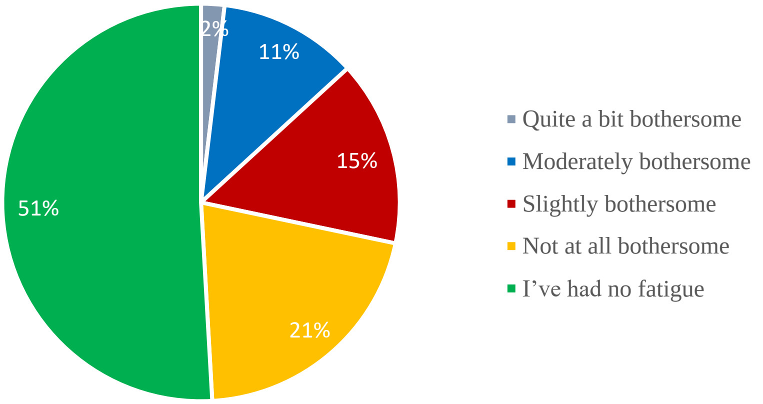

(7.) On average, how many times has **shortness of breath** limited your ability to do what you wanted?

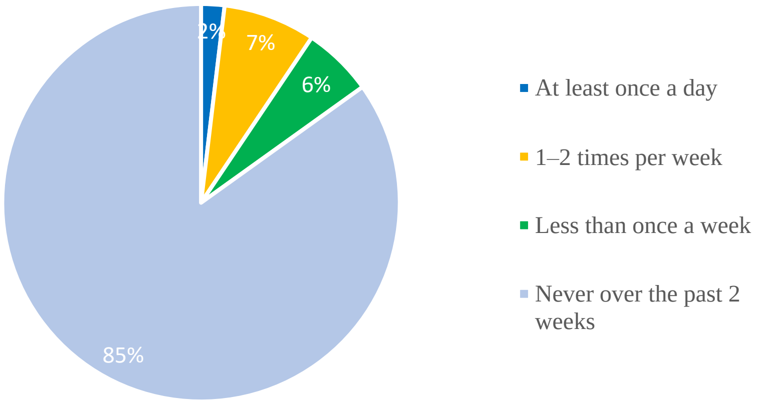

(8.) How much has your **shortness of breath** bothered you? It has been …

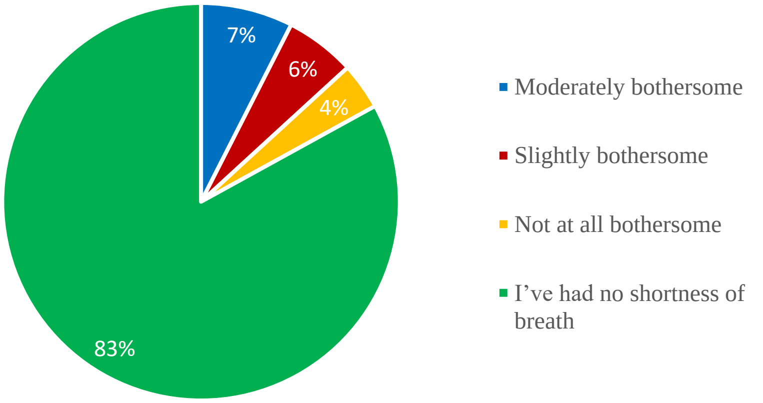

(9.) On average, how many times have you been forced to sleep sitting up in a chair or with at least 3 pillows to prop you up because of **shortness of breath**?

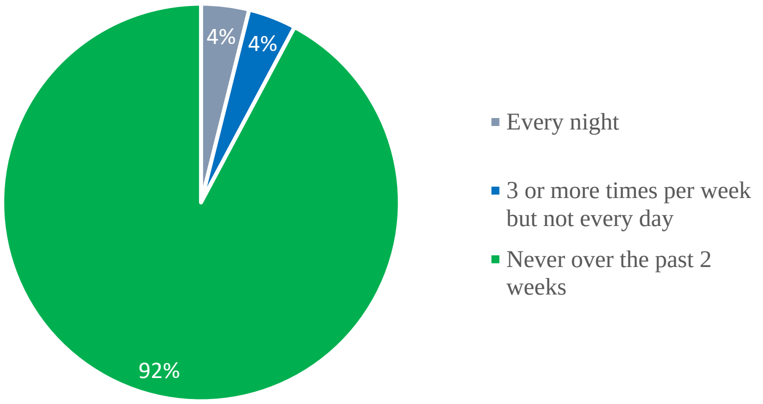

(10.) How much has your **heart failure** limited your enjoyment of life?

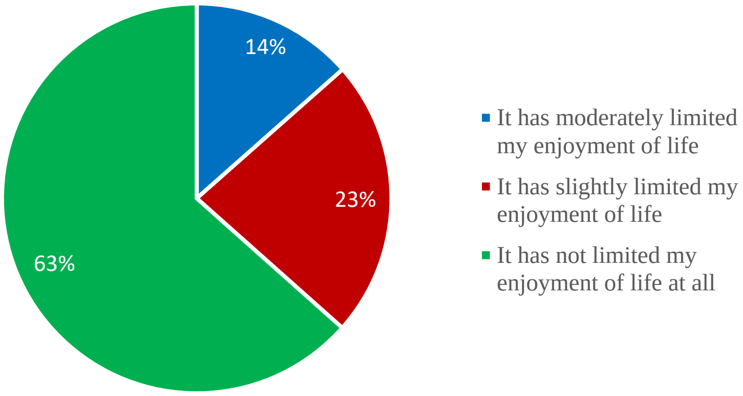

(11.) If you had to spend the rest of your life with your **heart failure** the way it was during the pre-COVID period, how would you feel about this?

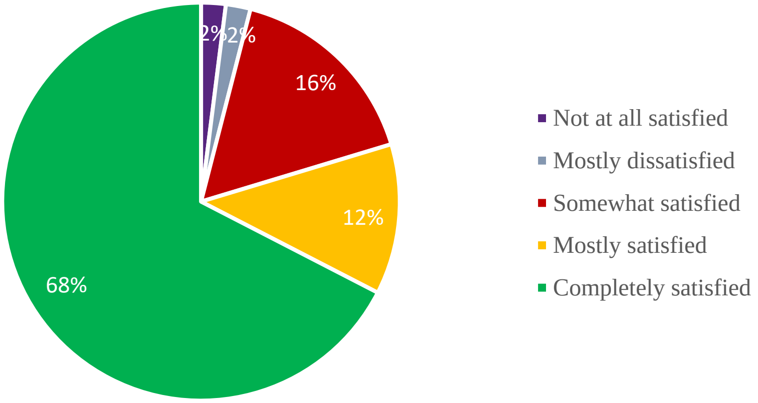

(12.) How often have you felt discouraged or down in the dumps because of your **heart failure**?

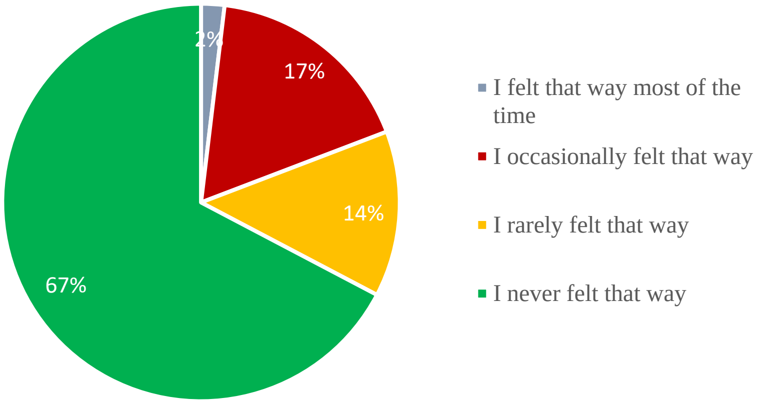

(13.) How much does your heart failure affect your lifestyle? Please indicate how your heart failure may have limited your participation in the following activities.

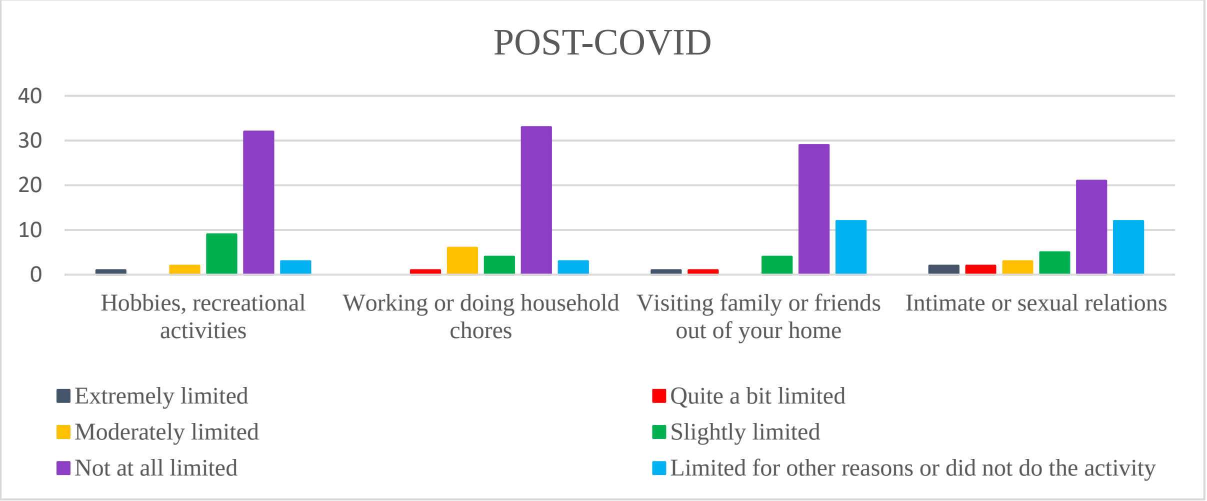

5-level EQ-5D descriptive system

Pre-COVID

(1.) Mobility

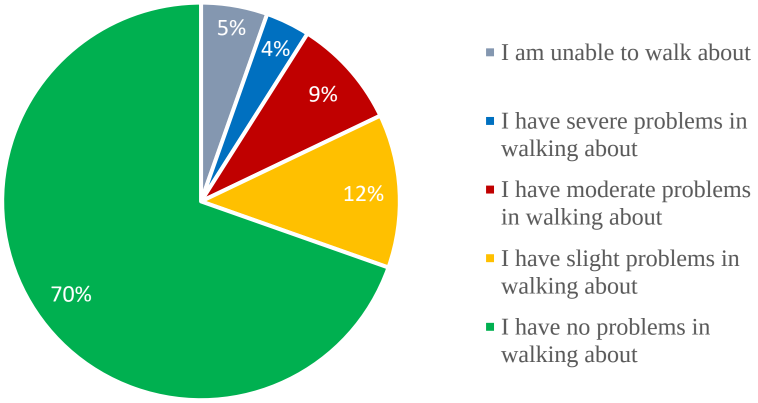

(2.) Self-care

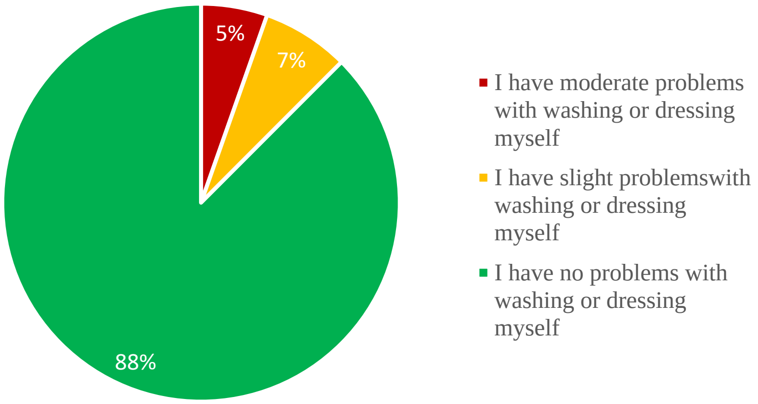

(3.) Usual activities (e.g. work, study, housework, family or leisure activities)

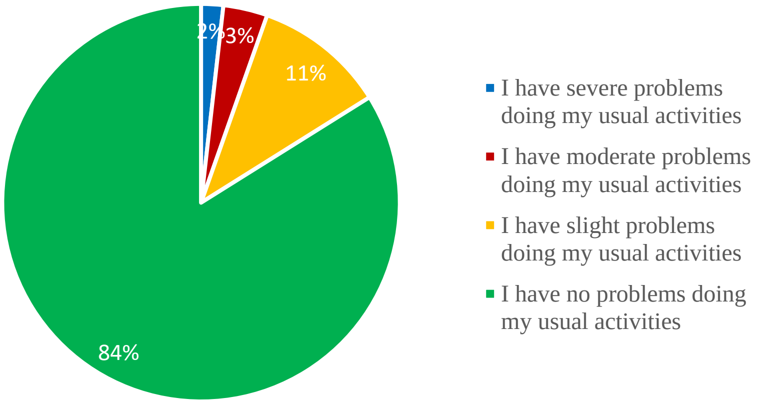

(4.) Pain/discomfort

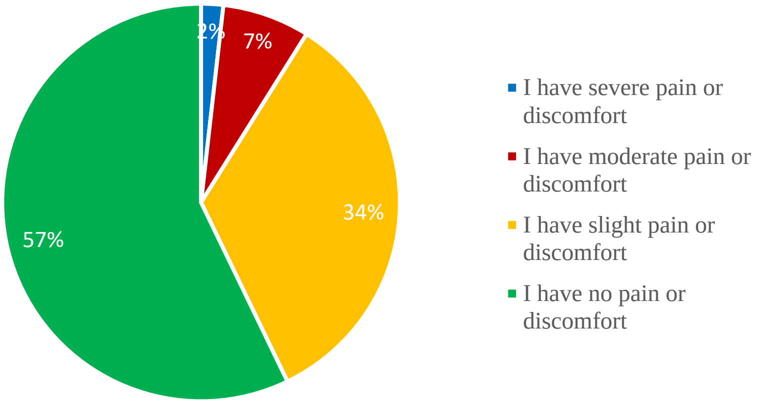

(5.) Anxiety/depression

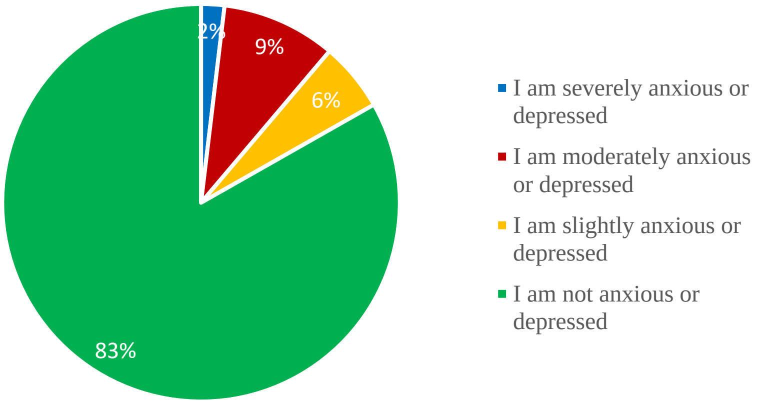

Lockdown

(1.) Mobility

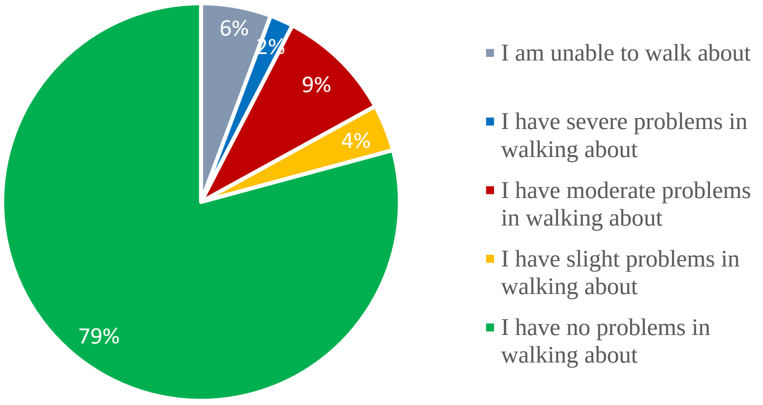

(2.) Self-care

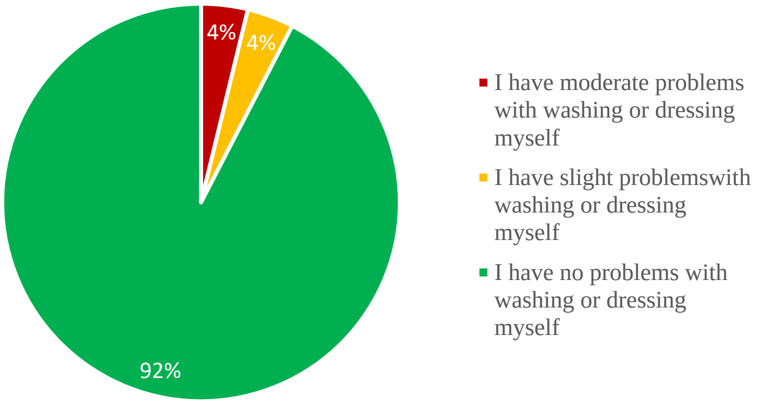

(3.) Usual activities (e.g. work, study, housework, family or leisure activities)

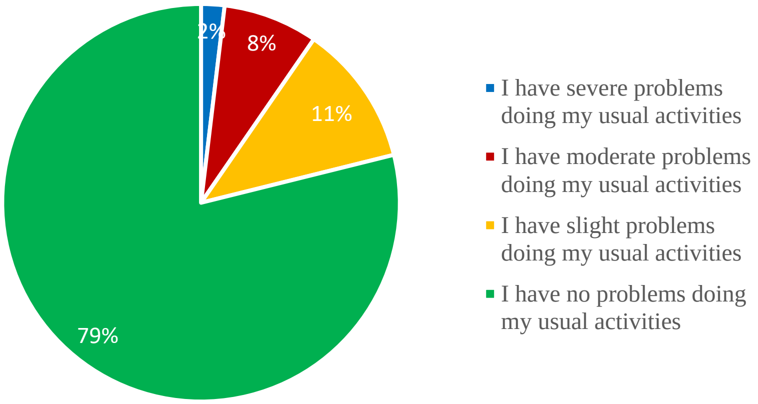

(4.) Pain/discomfort

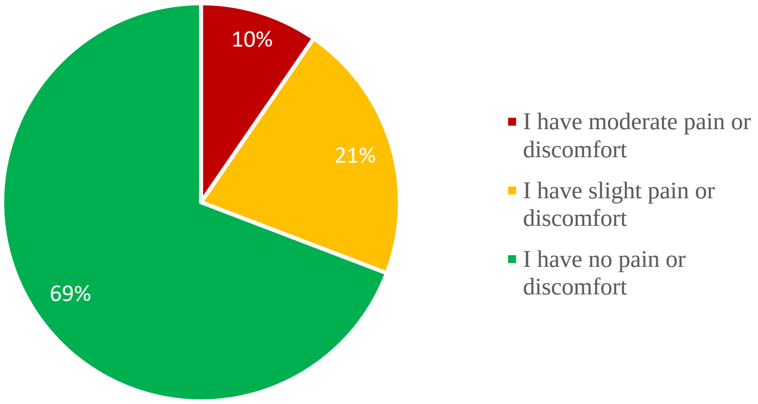

(5.) Anxiety/depression

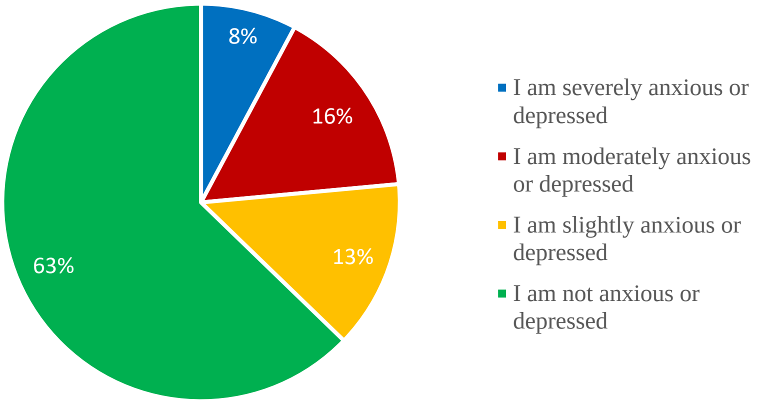

<u>Post-COVID</u>
(1.) Mobility

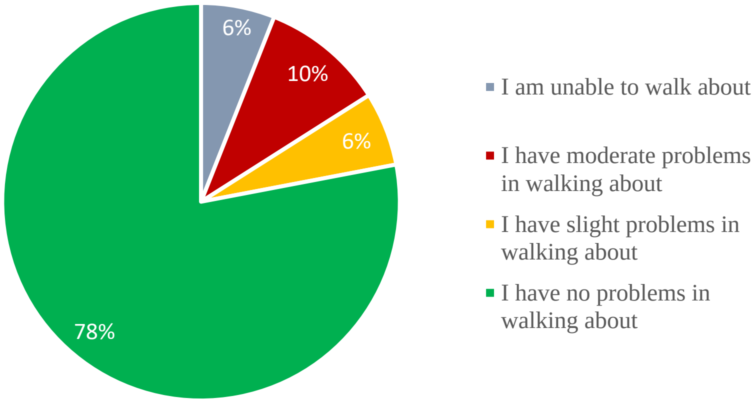

(2.) Self-care

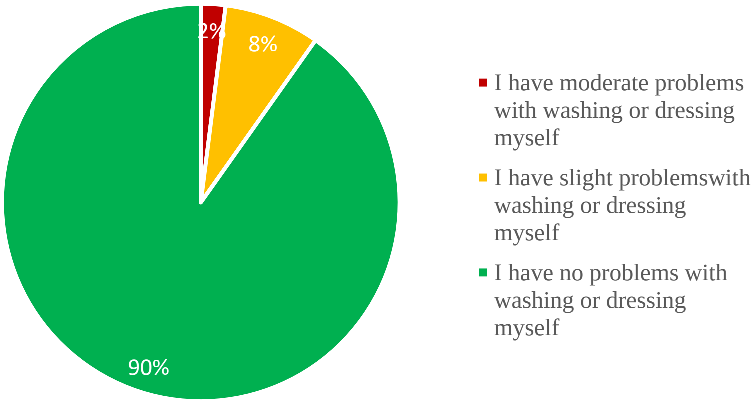

(3.) Usual activities (e.g. work, study, housework, family or leisure activities)

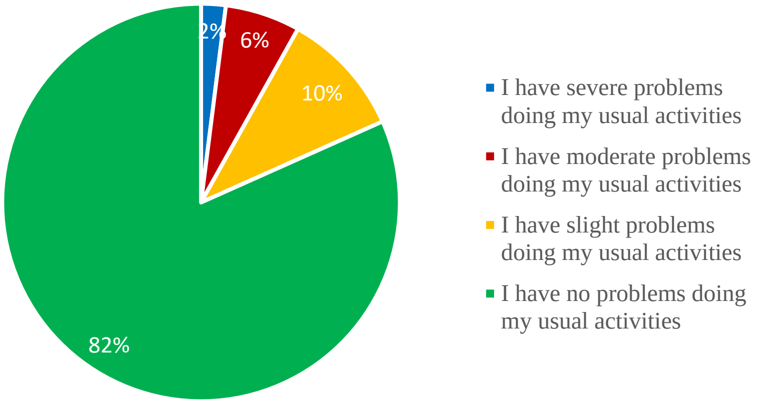

(4.) Pain/discomfort

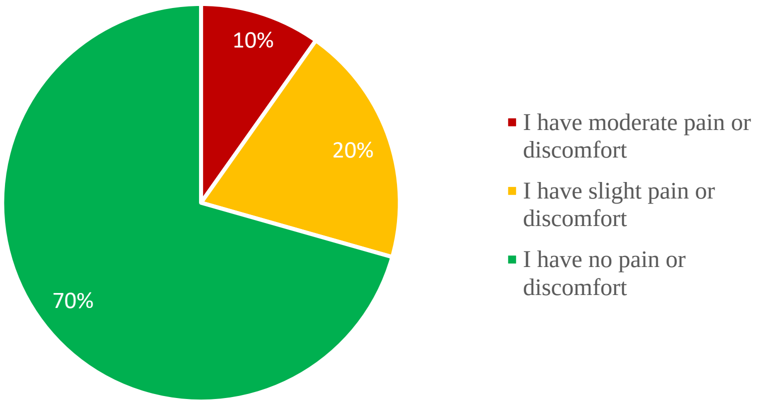

(5.) Anxiety/depression

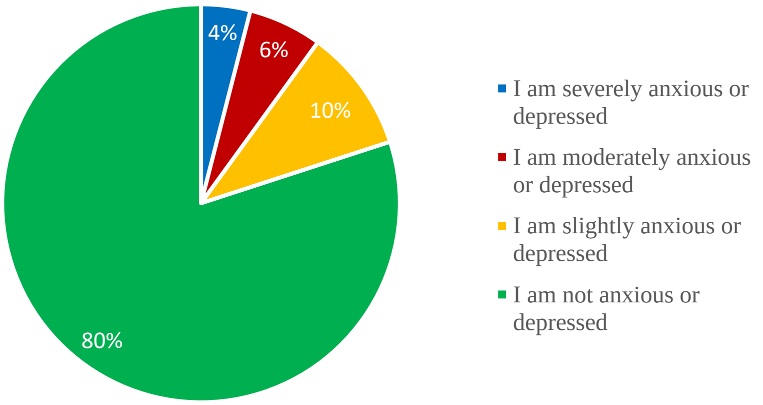

Health condition

Please rate your health on scale from 0 to 100, where 0 is the worst and 100 is the best.

In March

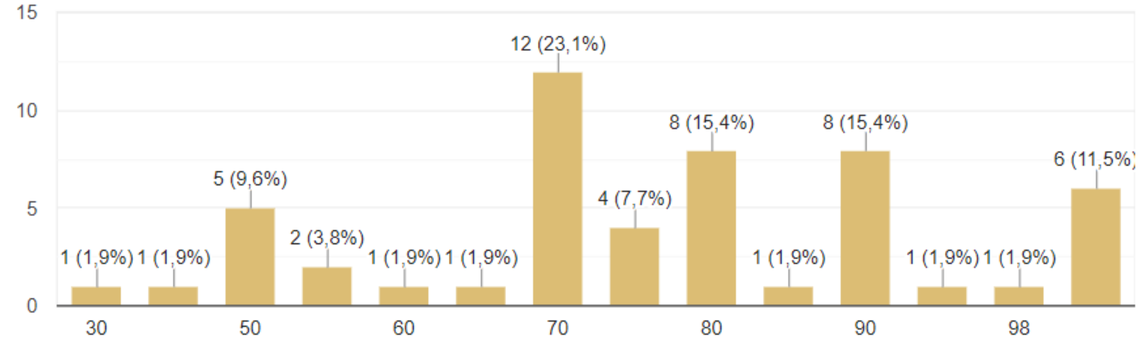

In April

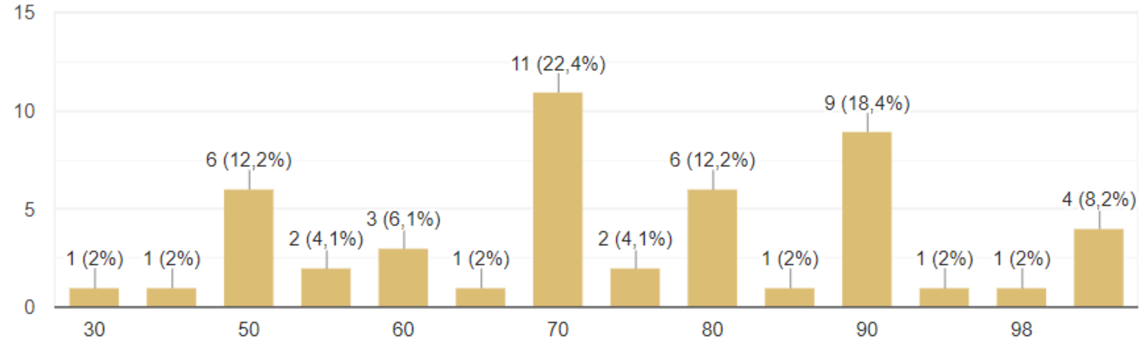

In June

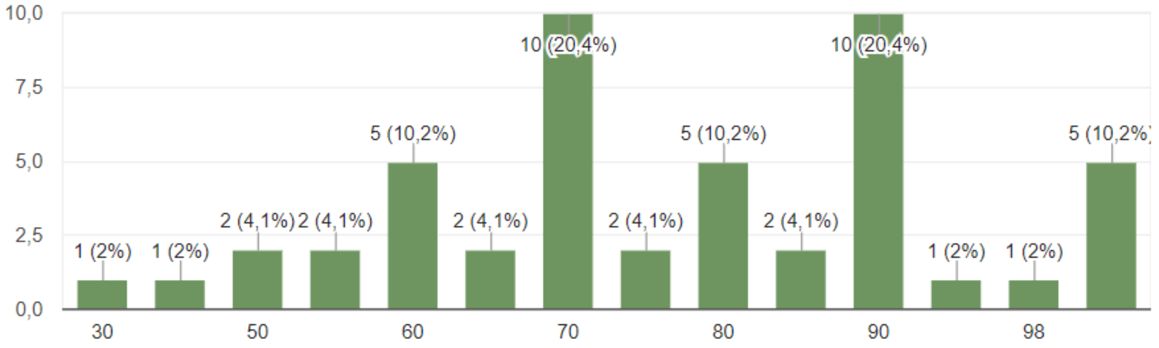

Supplement: Supplementary file 1 [file jcdd-10-00214-s001.zip › jcdd-2381506-supplementary.pdf]
